# Clinical Lectures on Diseases of the Eye

**Published:** 1864-03

**Authors:** E. L. Holmes

**Affiliations:** Chicago, Lecturer on Diseases of the Eye and Ear in Rush Medical College, and Surgeon of the Chicago Charitable Eye and Ear Infirmary


### CLINICAL LECTURES ON DISEASES OF THE EYE.

By E. L. HOLMES, M. D., of Chicago,

*Lecturer on Diseases of the Eye and Ear in Rush Medical College, and Surgeon of the Chicago Charitable Eye and Ear Infirmary.*

### THE CORNEA—ANATOMY AND PATHOLOGY.

Gentlemen: You have already seen a large number of patients with chronic conjunctivitis, in nearly all of whom there were secondary affections of the cornea. That you may understand these secondary affections of the cornea, I shall call your attention to the structure and diseases of this important portion of the eye, before finishing what I wish to say upon chronic inflammation of the conjunctiva and its treatment. I have stated that the diseases of the conjunctiva claim your first and most careful consideration, as being most common, and primarily the cause of more cases of blindness in the Northwest, than the diseases of any other part of the eye. It is scarcely less important for you to study well the cornea and its diseases, since it is by the destruction of a part or the whole of the cornea that conjunctivitis so often destroys vision.

The cornea is a circular membrane, composed of three principal layers, the middle of which, or the true cornea, is attached at its periphery to the sclerotic. The line of demarcation between the cornea and sclerotic is quite abrupt, but the fact, that the external layers of the sclerotic project slightly over the posterior layers of the cornea gives the appearance of indistinctness to the line of union. The manner in which the crystal of a watch is inserted in its rim is a good illustration of the relative position of the cornea and the sclerotic. The conjunctiva encroaches slightly upon the cornea, especially at its upper and lower portions. This rim—the limbus of the conjunctiva,—is sometimes, even in the young, so broad and opaque above and below, that the cornea presents a very decidedly oval form. The limbus conjunctival is exceedingly liable to become vascular in inflammations of the conjunctiva.

The limbus must not be confounded with the arcus senilis, which is a narrow grey ring running parallel with the periphery of the cornea, but situated within a narrow ring of transparent cornea.

Mr. Canton has written a monograph, showing the relation between a tendency to fatty degeneration and the arcus senilis. This change in the cornea may probably be regarded as normally incident to advanced age—like the presence of grey hairs, falling of the teeth, and presbyopia.

The thickness of the cornea is about half a line, being somewhat more at the periphery than at the centre, in the adult. The degree of convexity in the normal eye is nearly absolutely the same in all its diameters. Occasionally, the convexity is found to be more in one diameter (meridian) than in another, as you will learn, when you study the use of lenses in certain abnormal conditions of the refracting media.

The cornea is a very firm membrane, as is proved in cases of rupture ot the globe by simple pressure. The sclerotic almost invariably yields before the cornea.

Although it seem to be a perfectly homogenous, glassy membrane, it is quite complex in its structure, as is shown by the microscope and the action of certain reagents. It is composed of three layers: 1st, the epithelium, which is a continuation of the conjunctiva; 2d, the true cornea, a continuation of the sclorotic, and 3d, the serous membrane, which is in contact with the aqueous humor, and is a continuation of the outer layer of the choroid.

The true cornea is composed of fibrous tissue, resembling, in some respects, the ordinary areola tissue of other organs. In this tissue is found a large number of spindle and star shaped cells, with numerous minute tube-like appendices, ramifying in all directions, which last are supposed by some to be connnected with the process of nutrition. There are no true capillaries in the cornea, except at its periphery. The ciliary nerves are traced into the substance of the cornea in the form of delicate transparent nerve tubes.

The epithelium is composed of three layers, each of which is characterized by the peculiar appearances of its cells. The serous layer is composed of a structureless membrane, covered with epithelial cells, in direct contact with which latter is the aqueous humor.

It should be remembered, that in the fœtus, all these elements are quite opaque. As development progresses, they become less and less opaque, till, finally, they are united firmly into a transparent, nearly homogenous membrane.

The cornea is not only the window, as it were, by which light is simply admitted into one of the chambers of the brain; it is also one of the instruments, by means of which perfect images of external objects are formed upon the retina.

The cornea is subject to several very important diseases—nearly all of which are different phases of inflammation. A few words regarding their pathology will enable you to understand the manner in which they prove destructive to vision.

The first visible indications of inflammation in the cornea seem to be confined to the spindle and star shaped cells, and to the deeper cells of the epithelial layer. These cells and their appendices become filled with greyish granules, and much enlarged. They encroach upon the intercellular substance, which is absorbed or transformed into a fatty mass, mingled with pus cells; the tissue is destroyed. If the process is confined to the surface, the cells are cast off, leaving excoriations, or ulcers. If it is limited to the middle layers, abscesses are formed. An abscess, by the rupture of its outer walls, may be transformed into an ulcer.

This process, although it is the same in all cases of inflammation, is exceedingly variable, as regards its extent and rapidity of its course. It may be confined to a very minute superficial spot or spread over an extensive surface, causing a minute or extensive excoriation; if somewhat deep, a deep ulcer will be the result, varying according to the extent and depth of tissue affected.

Sometimes the diseased action will continue for months, without scarcely any change; or it may speedily terminate in the destruction of nearly the whole cornea. It is often difficult to explain these differences in cases in which all the conditions seem alike.

A very common affection of the cornea, and one exceedingly liable to accompany chronic inflammation of conjunctiva and "granulated" lids, is the so-called vascular cornea. Already in the earlier stages of acute conjunctivitis the fine capillaries of the limbus of the conjunctiva are observed to be enlarged, and filled with blood. The irritation produced by the constant rubbing of the roughened palpebral conjunctiva increases the inflammation. The changes in the spindle and star shaped cells take place as above described; their appendices anastomosing freely with each other, form a network of capillaries. A greyish colored exudation is also found in the superficial layers of the cornea, around these new vessels. This change usually takes place on the upper portion of the cornea, in consequence of the motion of the upper lid. Abrasions of the cornea, produced by burns, scalds, and other injuries, or repeated attacks of phlyctenulae, may also be the cause of the growth of vessels upon the cornea. As the vessels increase in number, and as lymph is deposited around these vessels, the surface of the cornea becomes roughened and red, presenting somewhat the appearance of "granulated" lids. This condition is known by the term Paunus, and may exist over a part or the whole of the cornea.

I have briefly explained the manner in which ulcers and abscesses are formed. Occasionally, the pus of the abscess is discharged through its posterior wall, the anterior wall remaining intact; in this case, the pus is discharged into the anterior chamber, forming what is called Hypopion. If the suppurative inflammative attacks the deeper layers of the cornea, the pus is sometimes diffused between its layers, (usually downwards) till it reaches the inferior line of separation between the cornea and sclorotic. In this case the pus renders the lower part of the cornea opaque, the opacity being convex at its lower border and irregular at its upper edge, and is called onyx, or unguis, from its resemblance to the lunula of the finger (thumb) nail.

The diseased action, as above described, especially the formation of ulcer, is induced by conjunctivitic. The disturbance of the process of nutrition, the pressure of granulations of the lids and particularly the pressure of the œdematous conjunctiva, encroaching upon the edge of the cornea, as it often does in acute conjunctivitis, induce ulceration in this important part of the eye.

But what is the final result of these abnormal changes? The ulcer, whether superficial or deep, may heal; leaving the cornea perfectly transparent. The contents of an abscess may be absorbed and the cornea left in its normal condition. And it is astonishing how speedily an ulcer, an onyx or a Hypopion will sometimes disappear, and how completely the cornea will resume its normal appearance.

But, unfortunately, the termination of these lesions is not always so favorable. The disease may become chronic, causing the patient much suffering and even loss of sight. The new tissue, which takes the place of the abscess or ulcer, instead of being transparent is too often opaque—hence opacities of the cornea. If the opacity is extensive or situated front of the pupil you can readily see how the rays of light are prevented from entering the eye.

Another unfavorable result of ulceration, is perforation of the cornea. The acqueous humor is then evacuated, and the iris and lens thrown forward in contact with the cornea. If the perforating ulcer is very minute and in the centre of the cornea, it may soon heal and the iris and lens be restored to their normal position, since the plastic lymph from the ulcerated cornea is deposited within the space occupied by the pupil without touching the iris and without binding the iris and cornea together. Occasionally in such a case the lymph will be thrown upon the centre of the anterior capsule of the lens, and we there have a small central opacity of the anterior capsule.

But if the perforation is near the peripheny of the cornea, the iris is very liable to form an adhesion with the cornea. Recovery with good vision may result. When the opening is extensive, a portion of the iris almost inevitably protrudes through it. Although vision may sometimes be retained, the inflammation of the iris and cornea is apt to cause softening of their tissues; as the perforation becomes filled with a portion of the iris and lymph, the softened cornea is no longer able to support the pressure outwards of the fluids of the globe and a projection occurs, called staphyloma. Not unfrequently the whole cornea from the effects of extensive ulceration or chronic inflammation, becomes softened and unable to support the pressure of the contents of the globe. The consequence is a large unsightly tumor, called total staphyloma of the iris and cornea. The walls of the staphyloma may be very thin, or they may be very thick and firm, in consequence of the organization of a large quantity of lymph within or upon the diseased tissues.

Sometimes the cornea, softened by inflammation, is pressed outwards in the form of a cone or of a hemisphere, and may retain its transparency. This condition is called corneal cornea. There is almost an infinite variety of form presented by the diseased cornea.

Before closing, I must repeat what I have before endeavored to impress upon your minds, that it is impossible for you to acquire skill in detecting the commencement of these changes in the cornea, without cultivating the habit of examining carefully the cornea, iris, pupil, and their relative normal position, in a large number of healthy individuals.

